# Transarterial chemoembolization for advanced hepatocellular carcinoma without macrovascular invasion or extrahepatic metastasis: analysis of factors prognostic of clinical outcomes

**DOI:** 10.3389/fonc.2023.1072922

**Published:** 2023-06-06

**Authors:** Ji Hoon Kim, Jin Hyoung Kim, Hyun-Ki Yoon, Gi-Young Ko, Ji Hoon Shin, Dong Il Gwon, Heung-Kyu Ko, Hee Ho Chu, Seong Ho Kim, Gun Ha Kim, Yonghun Kim, Shakir Aljerdah

**Affiliations:** ^1^ Asan Medical Center, College of Medicine, University of Ulsan, Songpa-Gu, Republic of Korea; ^2^ Ajou University Hospital, College of Medicine, Ajou University, Najran, Saudi Arabia; ^3^ College of Medicine, Najran University, Najran, Saudi Arabia

**Keywords:** hepatocellular carcinoma, chemoembolization, ethiodized oil, treatment outcome, Barcelona clinic liver cancer (BCLC) staging

## Abstract

**Objectives:**

To evaluate the safety and efficacy of TACE and factors predicting survival in patients with advanced hepatocellular carcinoma (HCC) without macrovascular invasion (MVI) or extrahepatic spread (EHS).

**Methods:**

This single-center retrospective study included 236 treatment-naïve patients who underwent TACE as first-line treatment for advanced HCC without MVI or EHS between January 2007 and December 2021.

**Results:**

Following TACE, the median overall survival (OS) was 24 months. Multivariate Cox regression analyses revealed that tumor number ≥4 (risk point: 3), maximal tumor size >10 cm (risk point: 2), Child–Pugh class B (risk point: 2), alpha-fetoprotein (AFP) concentration ≥400 ng/mL (risk point: 2), and presence of HCC rupture (risk point: 2) were risk factors significantly associated with OS. The expected median OS among patients with <2, 2–4, and 5–9 risk points were 72, 29, and 12 months respectively. The major complication rates were significantly lower in patients with maximal tumor size ≤10 cm than in those with maximal tumor size >10 cm (4% [5/138] vs 21% [21/98], p = 0.001).

**Conclusion:**

TACE may be safe and effective in selected patients with advanced HCC without MVI or EHS, with a median OS of 24 months. Patients with limited tumor burden, compensated liver function, absence of HCC rupture, and favorable biologic markers may benefit the most from TACE. TACE is not recommended for patients with huge HCCs (>10 cm) because of its high rate of major complications (21%).

## Introduction

The Barcelona Clinic Liver Cancer (BCLC) staging system is the most frequently used staging system for hepatocellular carcinoma (HCC), determining patient prognosis and suggesting treatment algorithms based on tumor-, patient-, and liver function-related factors (1). Advanced HCC (BCLC stage C) includes patients who have macrovascular invasion (MVI) and/or extrahepatic spread (EHS) and/or tumor-related symptoms, defined as an Eastern Cooperative Oncology Group (ECOG) performance status (PS) of 1 or 2 (2). The BCLC staging system is applicable to patients with highly heterogeneous clinical and oncological features. However, despite this heterogeneity, only systemic therapy is currently recommended as first-line treatment for patients with advanced HCC (1, 3–5).

Several recent studies, however, have suggested that patients with symptomatic HCC without MVI or EHS should not be regarded as having advanced-stage disease. Furthermore, patients with advanced HCC without MVI or EHS in a real-world setting have frequently been treated with locoregional therapy, such as transarterial chemoembolization (TACE) (6–10). The efficacy of TACE, however, has been insufficiently defined, and factors associated with prognosis in patients treated with TACE remain unclear. The clinical need for a more effective treatment strategy of patients with advanced HCC have made it necessary to determine the role of TACE in this patient group. The present study therefore evaluated tumor responses and factors predicting survival after TACE in patients with advanced HCC without MVI or EHS.

## Materials and methods

### Patient eligibility

The records of our institution were retrospectively searched to identify treatment-naïve patients who underwent TACE as first-line treatment for advanced HCC between January 2007 and December 2021. Advanced HCC was defined according to the BCLC staging system. Patients with HCC, with or without cancer-related symptoms (ECOG PS of 1 or 2), were included (1). Patients with MVI and/or EHS, those with prior or current malignancy other than HCC, and those lost to follow-up during the study period were excluded. The demographic and clinical characteristics of all patients, including age, sex, liver disease etiology, and biochemical parameters, were obtained at the time of HCC diagnosis. The study protocol was approved by the Institutional Review Board or our center, which waived the requirement for written informed consent.

### TACE procedure

TACE was performed through right femoral artery puncture and cannulation under local anesthesia. Superior mesenteric and common hepatic arteriographies were achieved with a 5-F catheter (Rősch hepatic catheter; Cook) to assess the direction of portal flow, the anatomy of the hepatic artery, tumor size and location, and feeding arteries. Patients underwent cisplatin-based TACE (2 mg/kg body weight) with a 1.7–2.4-F microcatheter (Progreat Lambda, Terumo, Tokyo, Japan; Renegade, Boston Scientific, Cork, Ireland; Carnelian, Tokai Medical Products, Aichi, Japan). A 1:1 emulsion of cisplatin and iodized oil (Lipiodol^®^, Laboratoire Guerbet) was infused into the feeding artery. Subsequent embolization was performed with gelatin particles (Upjohn, Kalamazoo, MI, USA) until arterial flow stasis was observed.

Patients were initially followed-up by contrast-enhanced CT or MR imaging and laboratory tests, 1 month after TACE. Subsequent follow-up examinations were repeated every 2–3 months during the first 2 years, and every 3–6 months thereafter until HCC recurrence. Repeated TACE was performed in patients with insufficient responses after a single session of TACE and those with recurrent tumors.

### Definition and data assessment

The primary outcomes were patient overall survival (OS, measured in months) and factors predictive of OS after TACE. OS was defined as the period between initial TACE and patient death. Pretreatment risk factors evaluated included the number of tumors (<4 vs. ≥4) (11), tumor size (≤10 cm vs. >10 cm) (12), morphological tumor type (nodular vs. infiltrative), tumor extent (unilobar vs. bilobar), presence or absence of portal hypertension, presence or absence of tumor rupture (13), Child–Pugh class (A vs. B), serum alpha-fetoprotein (AFP) concentration level (<400 ng/mL vs. ≥400 ng/mL) (14), presence or absence of underlying liver cirrhosis (LC), and presence or absence of bile duct invasion. Portal hypertension was diagnosed if the patient met any of the following criteria: noticeable portosystemic collaterals, ascites, esophageal varix, and splenomegaly with thrombocytopenia (platelet count <100,000/mm^3^) (15).

The secondary outcomes were progression-free survival (PFS), radiologic tumor response, and complications following TACE. PFS was defined as the time from initial TACE to tumor progression or death from any cause. Tumor response was evaluated according to the Modified Response Evaluation Criteria in Solid Tumors (m-RECIST), divided into four response categories: complete response (CR), partial response (PR), stable disease (SD), and progressive disease (PD) (16). Patients with CR or PR were categorized as tumor responders, and patients with SD or PD were categorized as nonresponders. Initial tumor response 1 month after TACE and the best tumor response over the entire study period were evaluated (17). Complications were categorized using the Society of Interventional Radiology (SIR) clinical practice guidelines. Major complications were defined as marked escalation of care (ie, hospital admission or prolongation of existing hospital admission for > 24 hours, hospital admission that is atypical for the procedure, inpatient transfer from regular floor/telemetry to intensive care unit, or complex intervention performed requiring general anesthesia in previously nonintubated patient), life-threatening or disabling event, or patient death (18, 19). All other complications were considered minor.

### Statistical analysis

Potential risk factors predicting OS after TACE were identified by univariable Cox-proportional hazards analyses. Variables with a p-value <0.05 were entered into a multivariate Cox-proportional hazards model using backward stepwise elimination and considering multicollinearity. A risk score was assigned to each variable based on its β regression coefficient in the multivariable Cox regression analyses, and the prediction model was designed according to risk scores. OS and PFS rates were estimated by the Kaplan–Meier method and compared using Log rank tests. Factors associated with major complications were evaluated by multivariable logistic regression analysis. Variables with p < 0.25 in the univariable analyses were entered into a multivariable analysis. All statistical analyses were performed using SPSS version 25 (IBM Corp.) and MedCalc Statistical Software version 20.1 (MedCalc Software Ltd).

## Results

### Patient characteristics

Of the 1256 patients who underwent TACE as first-line treatment for advanced HCC during the study period, 251 had advanced HCC without MVI or EHM. Fifteen of these patients were excluded from this study, 11 because they were lost to follow-up and 4 because they had a prior or concurrent malignancy. The study population thus consisted of 236 patients, 200 men, and 36 women, of mean ± SD, age 59 ± 12 years. Most patients were Child–Pugh class A (85%), had an ECOG PS of 1 (89%), had underlying liver cirrhosis (LC) (81%), and had nodular type tumors (82%). The major etiology of HCC was chronic hepatitis B (78%). Of the 236 patients, 18 (8%) had bile duct invasion, 40 (17%) had portal hypertension, and 40 (17%) had tumor rupture with perihepatic hematoma. The baseline characteristics of the included patients are summarized in **Table 1**.

**Table 1:** Demographic and clinical characteristics of the 236 patients included in the study.

Characteristic	Value
**Age (years)^†^**	58.7 ± 11.8
Sex	
** Men**	200 (85%)
** Women**	36 (15%)
Number of tumors	
** <4**	140 (59%)
** ≥4**	96 (41%)
Morphological tumor type	
** Nodular**	194 (82%)
** Infiltrative**	42 (18%)
**Bile duct invasion**	18 (8%)
**Tumor rupture**	40 (17%)
Etiology	
** HBV**	183 (78%)
** HCV**	13 (5%)
** Alcohol**	15 (6%)
** Others**	25 (11%)
**Portal hypertension**	42 (18%)
**Underlying LC**	192 (81%)
Child–Pugh class	
** A**	200 (85%)
** B**	36 (15%)
ECOG PS	
** 1**	211 (89%)
** 2**	25 (11%)
AFP	
** <400 ng/mL**	126 (53%)
** ≥400 ng/mL**	110 (47%)
**Albumin (mg/dL) ^†^**	3.5 ± 0.5
**Total bilirubin (mg/dL) ^†^**	1.1 ± 1.6

† Data are presented as mean ± standard deviation.

Otherwise, the data are presented as number (%).

### Factors and model predicting overall survival

By the end of the follow-up period, 163 (69%) patients had died. The median overall survival (OS) time after TACE was 24 months (95% confidence interval [CI], 20–28 months). The cumulative OS rates at 1, 3, 5, and 10 years were 75%, 35%, 22%, and 16%, respectively.

**Figure 1:**
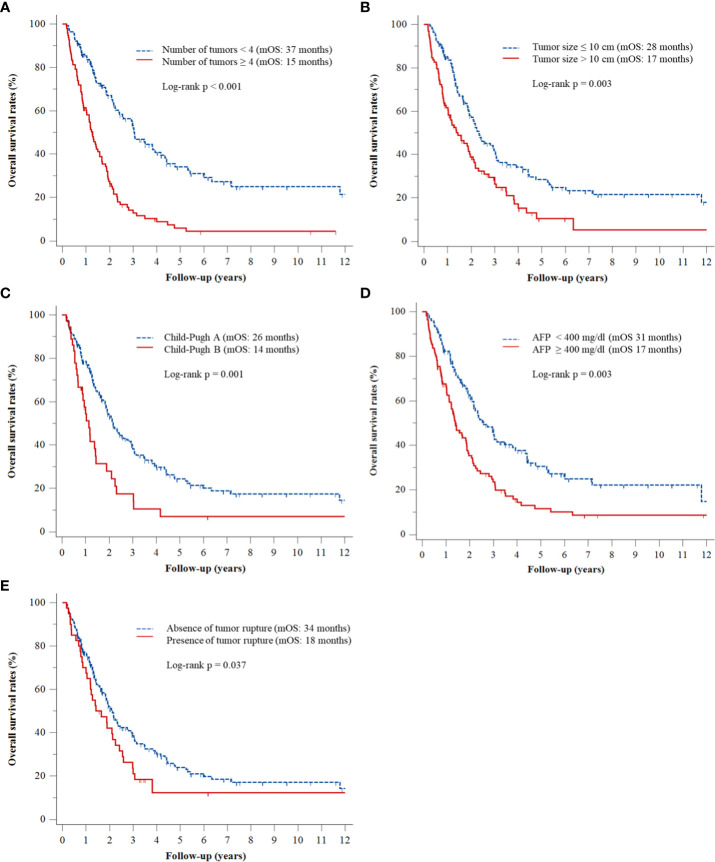
Kaplan–Meier analyses of OS rates according to **(A)** number of tumors, **(B)** tumor size, **(C)** Child–Pugh class, **(D)** serum AFP level, and **(E)** presence or absence of tumor rupture.

Multivariate Cox regression analyses revealed that ≥4 (vs. <4) tumors (adjusted hazard ratio [HR], 2.96; p < 0.001), tumor size >10 cm (vs. ≤10 cm) (adjusted HR, 1.63; p = 0.003), Child–Pugh class B (vs. class A) (adjusted HR, 2.12; p < 0.001), AFP concentration ≥400 ng/mL (vs. <400 ng/mL) (adjusted HR, 1.62; p = 0.003), and tumor rupture before TACE (vs. no tumor rupture) (adjusted HR, 1.55; p = 0.037) were significantly associated with OS rates (**Table 2**). The Kaplan–Meier curves of OS relative to these five factors are shown in **Figure 1**.

**Table 2:** Univariable and multivariable analyses of factors associated with overall survival after TACE in patients with advanced HCC without MVI and EHM.

	Univariable analysis			Multivariable analysis				
	HR	95% CI	*p* value	HR	95% CI	*p* value	Coefficient	Risk point
Tumor number (≥4)	2.27	1.20-3.45	<0.001^*^	2.96	2.13-4.12	<0.001^*^	1.01	3
Infiltrative tumor type	1.21	0.79-1.85	0.376					
Tumor size (>10cm)	1.56	1.11-2.18	0.010^*^	1.63	1.18-2.24	0.003^*^	0.49	2
Bilobar tumor involvement	1.24	0.83-1.86	0.289					
Ruptured HCC	1.56	1.02-2.38	0.039^*^	1.55	1.04-2.29	0.037^*^	0.44	2
Child-Pugh class B	2.01	1.23-3.28	0.005^*^	2.12	1.41-3.20	0.001^*^	0.75	2
Underlying LC	1.35	0.79-2.29	0.269					
Portal hypertension	1.26	0.80-2.00	0.315					
AFP > 400 ng/dl	1.62	1.17-2.23	0.003^*^	1.62	1.18-2.22	0.003^*^	0.48	2
Bile duct invasion	0.59	0.25-1.43	0.242					

HCC, hepatocellular carcinoma; MVI, macrovascular invasion; EHS, extrahepatic spread; HR, hazard ratio; CI, confidence interval; LC, liver cirrhosis; AFP, alpha-fetoprotein.

*Statistically significant.

**Figure 2:**
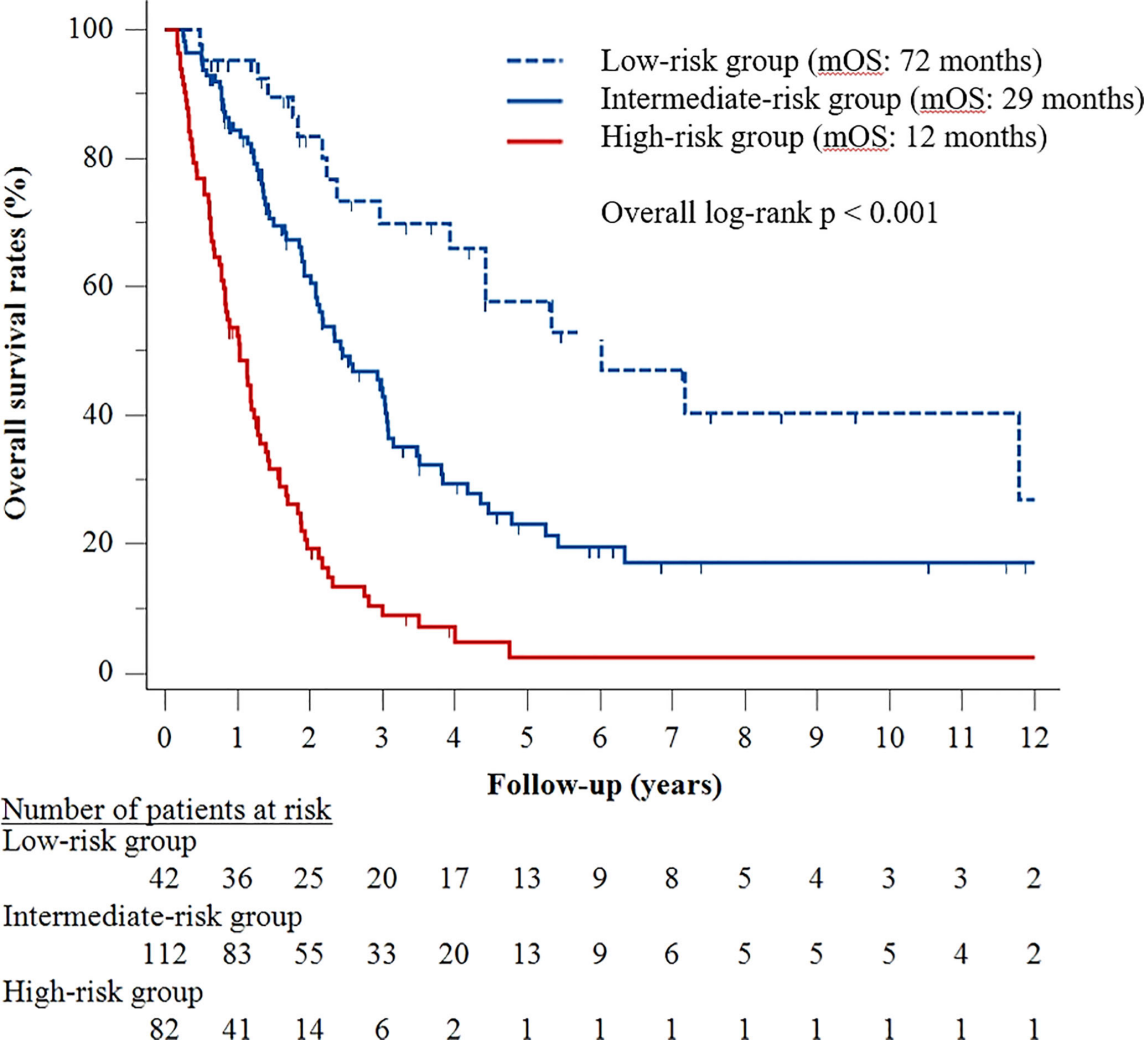
Kaplan–Meier curves for overall survival according to stratified risk groups.

A predictive model was subsequently developed based on the five factors identified in the multivariable Cox analysis. The presence of ≥4 tumors was assigned three risk points, whereas tumor size ≥10 cm, Child–Pugh class B, serum AFP ≥400 ng/mL, and tumor rupture were each assigned two risk points (**Table 3**). Patients were stratified by number of risk points into three groups, defined as low- (score <2), intermediate- (score 2–4), and high- (score 5–9) risk groups. Median OS was 72 months (95% CI, 34–110 months) in the low-risk group, 29 months (95% CI, 21–38 months) in the intermediate-risk group, and 12 months (95% CI, 9–16 months) in the high-risk group (p < 0.001) (**Figure 2**).

**Table 3:** Univariable and multivariable analyses of factors associated with progression-free survival after TACE in patients with advanced HCC without MVI and EHM.

	Univariable analysis			Multivariable analysis		
	HR	95% CI	*p*-value	HR	95% CI	*p*-value
**Tumor number (>4)**	2.25	1.58–3.20	<0.001^*^	2.34	1.73–3.16	<0.001^*^
**Infiltrative tumor type**	1.48	1.02–2.16	0.041^*^	1.40	0.97–2.03	0.070
**Tumor size (>10 cm)**	1.72	1.27–2.33	0.010^*^	1.75	1.31–2.34	<0.001^*^
**Bilobar tumor involvement**	0.97	0.69–1.37	0.860			
**Ruptured HCC**	1.24	0.87–1.78	0.249			
**Child–Pugh class B**	1.30	0.89–1.90	0.181			
**Underlying LC**	1.24	0.86–1.81	0.253			
**Portal hypertension**	1.21	0.84–1.74	0.300			
**AFP >400 ng/dl**	1.59	1.19–2.12	0.002^*^	1.61	1.21–2.13	0.001^*^
**Bile duct invasion**	0.27	0.38–1.31	0.271		

HCC, hepatocellular carcinoma; MVI, macrovascular invasion; EHS, extrahepatic spread; HR, hazard ratio; CI, confidence interval; AFP, alpha-fetoprotein.

*Statistically significant.

### Factors associated with progression-free survival

By the end of the follow-up period, 204 patients (86%) died or experienced HCC progression. The median post-TACE PFS of the 236 patients was 7 months (95% CI, 6–8 months). Multivariate Cox regression analyses revealed that covariates significantly associated with PFS included ≥4 (vs. <4) tumors (adjusted HR, 2.34; p = 0.001), tumor size >10 cm (vs. ≤10 cm) (adjusted HR, 1.75; p < 0.001), and AFP concentration ≥400 ng/mL (vs. <400 ng/mL) (adjusted HR, 1.61; p = 0.001) (**Table 2**).

### Tumor response and major complications

All 236 patients were evaluated by dynamic CT or MRI for tumor response 1 month after TACE, with 42 patients (18%) achieving CR, 126 (53%) achieving PR, 15 (6%) having PD, and 53 (22%) having SD. Thus, the initial tumor responder rate (CR plus PR) was 71%. During the follow-up period (median, 65 months; interquartile range, 31–114 months), 89 patients (38%) achieved CR, and 100 (42%) achieved PR, whereas 14 (6%) had PD, and 33 (14%) had SD as the best overall tumor response. The best overall tumor responder rate was 80%.

Major complications were observed in 26 (11%) patients after TACE. The major complications included acute renal failure in six patients; liver abscess in five; tumor lysis syndrome, acute cholecystitis, and persistent fever in three patients each; spontaneous bacterial peritonitis in two patients; and hepatic failure, allergic reaction to cisplatin, pleural effusion, and spinal infarction in one patient each. One patient died of tumor lysis syndrome (mortality rate, 0.04%). Major complication rates were significantly lower in patients with maximal tumor size ≤10 cm than in those with maximal tumor size >10 cm (4% [5/138] vs 21% [21/98], p = 0.001). Multivariate logistic regression analysis showed that tumor size >10 cm (odds ratio, 5.72; p = 0.001) was the only significant risk factor for major complication (**Table 4**).

**Table 4:** Univariable and multivariable analyses of pretreatment factors associated with major complications.

	Univariable analysis			Multivariable analysis		
	OR	95% CI	*p*-value	OR	95% CI	*p*-value
**Tumor number (≥4)**	0.78	0.31–1.95	0.595		
**Tumor size (>10 cm)**	7.98	2.79–22.80	<0.001	7.26	2.63–20.02	<0.001^*^
**Child–Pugh class B**	1.04	0.30–3.57	0.945		
**AFP >400 ng/dl**	0.49	0.30–1.59	0.118	0.52	0.21–1.24	0.115
**Ruptured HCC**	1.73	0.58–5.13	0.326		
**Bile duct invasion**	Nonestimable^a^				

OR, Odds ratio.

a Calculation was nonestimable because major complications did not occur in patients with bile duct invasion.

Factors with p < 0.25 in the univariable analysis were entered into a backward elimination multivariable logistic regression analysis.

## Discussion

To our knowledge, this study is the first to specifically investigate the survival outcomes and safety of first-line TACE in treatment-naïve, symptomatic HCC patients without MVI or EHS. First-line TACE showed acceptable efficacy in prolonging survival in these patients, with a median OS after TACE of 24 months. This median OS was longer than that observed in patients with advanced HCC treated with systemic treatment in the recent randomized phase III trials (median OS; 15 – 16 months) (20, 21). The median OS of patients with advanced HCC in the present study was similar to that of patients with intermediate HCC receiving TACE, which has been reported to range 25–30 months (22–25). Based on the extended indications for TACE and the suboptimal responsive to systemic treatment in this patient group, appropriate locoregional tumor control with TACE may be a better option than systemic treatment. In addition, the determination of tumor-related symptoms is frequently subjective as it is dependent on several confounding factors, including advanced age, LC, extrahepatic comorbidities, and individual sensitivity to pain (26). Thus, classifying patients as having advanced HCC based only on clinical findings, despite favorable oncological features, may be a limitation of the BCLC staging system. The results of this study indicate that symptomatic HCC patients without MVI or EHM should not be regarded as having advanced-stage tumors as it may limit any potential locoregional treatment.

Multivariable Cox regression analyses showed that pretreatment oncological features, including ≥4 tumors, tumor size >10 cm, and tumor rupture, as well as clinical factors, such as decompensated liver function and AFP ≥400 ng/mL, were statistically significant predictors of poorer OS. Because this study included a relatively large number of patients (n = 236), these five parameters could be used to develop a prediction model. Three risk groups were identified and classified as low-, intermediate-, and high-risk groups, with median OS of 72, 29, and 12 months, respectively. Based on this prediction model, the low-risk group, consisting of patients with <4 tumors, tumor size ≤10 cm, absence of tumor rupture, compensated liver function, and AFP <400 ng/mL, could benefit most from TACE. Therefore, TACE may be actively recommended as a preferable treatment option for this group.

The present study also showed that tumor rupture was associated with poor prognosis after TACE. Previous studies have yielded conflicting results on whether tumor rupture is a poor prognostic factor and on the role of TACE in patients with tumor rupture (27, 28). Based on recently reported data on equivalent survival outcomes in patients with ruptured HCC, the Sixth edition of the Liver Cancer Study Group of Japan (LCSGJ) guidelines does not consider tumor rupture in T staging. In contrast, the Eighth edition of the American Joint Committee on Cancer guidelines has classified HCC rupture as T4 stage (29). The results of the present study indicate that ruptured HCC may have prognostic significance in predicting a poorer OS. However, based on the management of ruptured HCC, TACE may be the best option for obtaining hemostasis for bleeding from a ruptured HCC as well as for antitumor treatment of the HCC itself. A recent meta-analysis (30) involving 21 studies reported that TACE was significantly superior to emergency surgery in reducing complication rates, with comparable 1 year survival rates. Therefore, TACE should not be precluded for the treatment of patients with ruptured HCC.

Although bile duct invasion (BDI) is regarded as an indicator of poorer prognosis in patients with HCC, few studies to date have specifically focused on HCC patients with BDI. Moreover, of the various HCC staging systems currently used, only the LCSGJ staging system regards BDI as having an impact comparable to that of MVI (31, 32). Interestingly, the present study found that BDI was not significantly associated with OS after TACE. Theoretically, HCC with BDI may be accompanied by portal vein tumor thrombus, which is considered prognostic of poorer OS. Because the present study excluded the features of portal vein invasion, BDI itself was not associated with survival outcomes. These findings suggest that TACE can be recommended to treat HCC patients with BDI without portal vein tumor thrombus.

In the present study, evaluation of safety profiles showed that only large tumor size (>10 cm) was significantly associated with the occurrence of major complications, with a relatively high rate (21/98; 21%). TACE has been considered a relative contraindication for the treatment of huge HCC (> 10 cm) due to the increased risk of peri-procedural adverse effects and the suboptimal treatment response (33, 34), and recent studies reported that hepatic arterial infusion chemotherapy (HAIC) was found to prolong OS compared with TACE in patients with huge HCC (35–37). A recent randomized phase III trial showed that the use of combinations of chemotherapy fluorouracil/leucovorin/oxaliplatin in HAIC group had a better OS (HR 0.57 [95% CI, 0.44 to 0.74]; P <.001) and a lower incidence of serious adverse events (19% vs. 30%, P = .03) compared with the TACE group for patients with large unresectable HCC without vascular invasion or extrahepatic metastasis (38). Thus, due to the high risk of major complications, TACE should not be recommended as a primary therapeutic option for patients with huge HCC. Additional randomized controlled trials are needed to determine a safe and effective option for the treatment of huge HCCs.

The optimal use of TACE as a treatment option for patients with Child-Pugh class B patients who are not eligible for liver transplantation remains uncertain. In real-world allocation, there is variation in the application of TACE due to variable angiographical techniques (lobar, selective, and super selective) and different chemo therapeutic drugs. According to the American

Association for the Study of Liver Diseases guidelines, TACE is recommended for asymptomatic patients with Child–Pugh class A. On the other hand, the European Association for the Study of the Liver guidelines expand the indication to include Child–Pugh class B7 patients without ascites while the Japan Society of Hepatology guidelines also extend the indication to Child–Pugh class B patients (39–41). In the present study, of 36 child-Pugh class B patients, majority of patients had a B7 score (n = 22, 61%), whereas 7 (19%) and 7 (19%) patients had B8 and B9 scores. Although indirect comparisons should be undertaken with caution, the median OS of Child-Pugh class B patients after TACE in this study was longer compared with results of a recent multicenter phase I/II trial investigating of immunotherapy in patients with Child-Pugh class B advanced HCC (14 months vs. 7.6 months, respectively) (42). However, the findings of the present study showed that Child-Pugh class B patients may have prognostic significance in predicting a worse OS outcome. Moreover, subgroup analysis for the Child-Pugh class B group was not feasible due to small size. Therefore, further studies are necessary to provide reliable data on the appropriate indications of TACE in patients with Child-Pugh class B patients

In this study, 29% (69/236) of patients were nonresponders. Close monitoring of liver function is mandatory for this group of patients because recent studies suggest that patients without a radiological response after TACE are likely to be ineffective and harmed by additional local therapies and may also be at risk of cumulative liver injury (43). This could lead to a deterioration of liver function and make patients ineligible for subsequent systemic treatment (4, 24, 44–46). Therefore, it is crucial not to miss the appropriate time for switching to therapy in patients with refractoriness to TACE.

The present study had several limitations. First, its retrospective, single-institution design may have a potential for selection bias. However, efforts were made to minimize potential bias by performing multivariate analyses with relatively a large sample size (n = 236). Second, we examined patients underwent only TACE as first-line treatment. Nowadays a lot of research has been made in adjuvant immuno therapy after TACE in advanced-stage HCC patients (47–52). Combining TACE with immunotherapeutic agents hypothesized that prolonged survival could be achieved because adjuvant immunotherapy can control TACE-induced neo-angiogenesis and reduce the risk of tumor recurrence and metastatic growth. So, prospective, multicenter randomized controlled trials comparing several therapeutic modalities are needed to optimize treatment in this specific group of patients.

In conclusion, TACE may be safe and effective in selected patients with advanced HCC without MVI or EHS, with a median OS of 24 months. Patients with a limited tumor burden, defined as <4 tumors and maximal tumor size ≤10 cm, compensated liver function, absence of HCC rupture, and AFP concentration <400 ng/mL, were found to benefit most from TACE. Because of its high rate of major complications (21%), TACE should not be recommended for patients with huge (>10 cm) HCCs.

## Data availability statement

The raw data supporting the conclusions of this article will be made available by the authors, without undue reservation.

## Ethics statement

Written informed consent was not obtained from the individual(s) for the publication of any potentially identifiable images or data included in this article.

## Author contributions

Conceptualization, JHK. Methodology, JHK. Validation, JHK and JS. Formal analysis, JK, and JHK. Data curation, JK, JHK, H-KK, HC, SK, JS, DG, G-YK, H-KY, YK, GK, and SA. Writing—original draft preparation, JK. Writing—review and editing, JHK, H-KK, HC, SK, JS, DG, G-YK, H-KY, YK, GK, and SA. Visualization, JK. Supervision, JHK. All authors contributed to the article and approved the submitted version.
